# Remarks on the Importance of Good Health in Ministers of the Gospel, and on the Best Means of Attaining It

**Published:** 1845-04

**Authors:** William M. Boling

**Affiliations:** Montgomery, Alabama


### Art. II.—*Remarks on the Importance of Good Health in Ministers of the Gospel, and on the best means of attaining it.* By William M. Boling, M.D., of Montgomery, Ala.

With all classes of men, in whatever pursuit engaged, whatever their calling, or whatever the duties devolving on them through life to perform, that these duties should be well and efficiently performed, the possession of good health on their part would seem to be almost absolutely necessary. Not that a man of superior ability or skill in his pursuit, may not, with but a very moderate degree of health, as effectually perform the duties of his station, as could one of but ordinary capacity, in the enjoyment of the most excellent health, and therefore, it may be said, perform them efficiently, but make the bodily health of the two equal, and the result will be different. Is it not reasonable to suppose that he, who while afflicted with bodily infirmities, performs his task as well as another in health, would be capable, his infirmities removed, to perform it better? If the steward with five talents, hides four of them in the ground, is the amount of interest reaped by his Lord greater than that from the servant who has but one? Is the amount of benefit derived from the ministerial functions of a man of the most brilliant intellect, but with that intellect clouded, soured, and overshadowed by bodily suffering, and the sphere of his active usefulness circumscribed by physical imbecility, greater than that derived from the active and healthy exercise of a mind of ordinary capacity and attainments, in the performance of like duties? That superiority of intellect is not unfrequently found in connection with great bodily infirmity no one will deny, and it would be absurd to argue, that, in the mass, men's intellects were to be judged by their physical powers alone. We would not expect from him whom nature has endowed with but common comprehension, in any state of corporeal health, an "Essay on man," a "Childe Herold," or a "Hamlet," though, should he make the attempt, we would expect him more nearly to attain success, than if, over the small amount of intellect, by nature allotted, disease and physical suffering were to throw their beclouding veil—that, cæteris paribus—the man in health, would always more efficiently accomplish his object, or peform his task, than one in a different state.

It is a fact of almost daily observence, that the manifestation of superior mental abilities is not incompatible with much physical weakness and with a greatly impaired state of bodily health, and hence it has been argued by some, that these conditions were favorable to the full development of the mental faculties. In favor of this argument, many bright examples are adduced from authors on all subjects—but principally from among the poets—and on the list, Pope and Byron are conspicuous. Of the former it may be conceded, that originally his constitution was feeble, and that his mind was of a superior order, but does this prove, that with good bodily health he would not have thought as well, or better? But it may be even questioned, if to this originally feeble constitution, his sedentary habits, the necessary consequence of his pursuit in life, did not add their enervating influence, and thus, that still further impaired health, which was in reality an indirect consequence of his genius, receive the credit of being measurably its cause? In many things, the proper connection of cause and effect is frequently reversed.

Of the latter, instead of being originally of a delicate constitution, it may be affirmed, on the contrary, if we may judge by what he himself says of his ability to undergo bodily fatigue, that he possessed one of the strongest; and though in person not large, he must have been unusually active and athletic. True, in after years, the consequence of every species of irregularity, of successive days of abstinence, preparatory to others of repletion, and perhaps a too free indulgence in wine and other intoxicating drinks, the profuse use of tobacco, and then again an injudicious system of medication for the purpose of relieving promptly, though temporarily, the system from the effects of these excesses, his health did become impaired, and under this state of impaired health he continued to write well,—better than in earlier life, when in better health. But does this prove any thing? Is it strange that the matured intellect of the man should think more strongly, or more correctly, than that of the Harrow school-boy? Is it strange that the boy should not give utterance to passions, pangs and disappointments felt only by the man? Should not clothe with language, wrongs and injuries, real or imaginary, endured only by him? Should not describe, with graphic accuracy, with poetic fire, scenes which he had not witnessed, dramas in which he had played no part? And here again, may we not say, that that genius and poetic fervor which prompted him to write, and to the neglect of the proper amount and regularity of exercise, necessary to the healthy performance of the bodily functions, were in part the cause of that impaired health, of which they have been supposed the consequence.

A word or two of Burns. Through some most excellent pieces by this author, there seems a strain of melancholy despondency, and of hopeless, cheerless gloom, the conception of a mind on which physical suffering had stamped its impress. In others, there is apparent a tincture of spite, of discontent and peevish sarcasm, the effusions of a mind 'sicklied o'er' by the perverting power of the demon dyspepsia. But, as works of genius, though much admired, are the 'Ode to Despondency,' 'Man was made to mourn,' and others of that character; and 'Holy Willie's Prayer,' and others of the same stamp, superior—are they equal, to that beautiful poem, pervaded by feeling of continued happiness, of religious satisfaction, the evidences of at least temporary bodily health, and of a mind at ease, unbiased by sympathy with corporeal suffering—'The Cotter's Saturday night?'

The performance of 'high and noble deeds,' the achievement of great victories, and the accomplishment of mighty revolutions, have often, it is said, been the work of men of feeble and sickly frames. But is this true? Does the diminutive statue of Alexander necessarily imply a sickly constitution; or must the bodily deformity of Gloucester, admitting it to be a historical fact, of which, however, there seems to be some doubt, be necessarily associated in our minds with bodily suffering and infirmity? But admitting these, and a few others, for the sake of argument, to be examples of what they are arrayed to prove, does it follow that the same spirits might not have accomplished as much, in some instances more, if associated with better bodily health; or that the sickly and infirm tenements, added fire and energy to the workings of the mind? Are they not indeed a few exceptions brought hastily forward to constitute a rule?

Although it has occasionally happened, that persons, apparently of delicate constitutions, and who have suffered much from bad health, have attained to good old ages—it is nevertheless a fact, as a general rule, that the vigorous frame outwears the more delicate machine, and the number of years attained, may be taken as a measure of the original strength of constitution, and the amount of tax imposed upon it by disease. This being admitted, if it should appear as a general rule, that those persons who have given evidence of superior mental endowments, have been long lived; it goes far to prove the importance of good bodily health to vigorous intellectual exertion. In any calculations of this nature, it would be proper to make allowances for early deaths, from acute diseases, in consequence of exposure necessarily attendant upon any particular pursuit; and for violent deaths upon the field of battle, and from other causes. It is evident, that among literary characters, such accidents are of less frequent occurrence than among most others, and least of all among females—among them then, would it be proper to look for evidence in such an investigation. The following list, comprising the principal female authors of distinction of the last century, which I find in an old number of the Quarterly Review, shews that these distinguished ladies lived considerably beyond the average duration of human life. Thus:

Lady Russell died at the age of 87, Mrs. Rowe at 63, Lady M. W. Montague 73, Mrs. Centliver 44, Lady Harvey 70, Lady Suffolk 79, Mrs. Sheridan 47, Mrs. Cowley 66, Mrs. McCauley 53, Mrs. Montague 81, Mrs. Chapone 75, Mrs. Lennox 84, Mrs. Trimmer 69, Mrs. Radcliffe 60, Mrs. Barbauld 63, Mrs. Delany 93, Mrs. Inchbald 68, Mrs. Piozzi 80, Mrs. Hannah More 88, Mrs. Hamilton 65.

In these few remarks, if I have rendered apparent, I will not say proved, that for man to perform his allotted duty here to the extent of which his mind may be capable, the possession of good bodily health is essential; and that by it power, clearness, and determination are added to his mental manifestations, the sphere of his usefulness thereby augmented, it is palpably apparent that the relative importance to the world in general of the good health of any particular class of men depends upon the usefulness of that class to their fellow-men, the effect the efficient performance of their part may have for the good, the effect its malperformance may have in the injury of mankind, may add to or detract from the happiness and comfort of man here, or may well, or ill prepare him for that "bourne whence no traveler returns."

*The importance of good health in Ministers of the Gospel.*— That which is so self-evident, needs little argument in its support. The paramount importance of their ministerial admonitions, instruction, advice and example, to the comfort, dignity, and well being of man here, and to his happiness hereafter, must be admitted by all who think, observe, or reason on the subject. In so far as man's future destiny should take precedence of all other considerations, just so far is it important above all other classes of men, that the individuals recognised in all countries, and in almost every age as the agents, to warn, prepare, and guide—to direct men's steps along the narrow path to peace; to teach them to avoid the shoals and quicksands on which their future weal may be wrecked, should have few physical ills of their own to occupy their thoughts—to mar the healthful workings of their minds, or to constrain or circumscribe their field of labor.

In times of pestilence, to whom with anxious eye does the dying man look for comfort, for a gleam of hope, sure that though all others fly him, he will not be wanting? Side by side with the physician, is seen the pious and benevolent clergyman, visiting the abode, where death stares him in the face, seeking to supply consolation in that trying hour. In the performance of these voluntary acts of benevolence and christian kindness, how much more efficient the vigorous, healthy man, than another less fortunately constituted.

One of the most important conceptions for the improvement of the moral and physical condition of man—one which has been the means of rescuing thousands from, of preventing millions from falling into a state of physical wretchedness and misery, and of moral degradation below even that of the brute—the Temperance cause; has it not mainly been carried out by the exertions of the clergy? Witness the benevolent exertions of Father Mathew in Ireland. It is true no one individual in this country has effected so much. The cause is obvious. There, he had the whole field to himself—here, thousands of willing hands have been extended to its cultivation.

That man should think charitably of, and act kindly towards his fellow man, that he should be lenient to another's faults, and ready to accord him all he merits—it is essential that the better feelings of his nature should be unmarred, his sense of right and charity, and all his kindlier sympathies unperverted by the blasting influence of bodily suffering over mental health. If this be true with regard to the mass, how much more important is it that they who should stand as 'bright examples,' whose conduct is looked to closely, whose thoughts and actions are severely scrutinized, and each of whom gives a bent, for much or little good, in proportion as he bears the test, in the immediate circle in which he moves, and of whom any gross error, throws scandal and disgrace upon his Maker's cause, far beyond it—should be at peace with all the world—should think and speak with charity of all! I once heard a talented but somewhat eccentric professor, in addressing his class, express himself in this emphatic manner, when speaking of a certain article of the Materia Medica, the power of which, in correcting derangements of the abdominal viscera, and alleviating dyspeptic affections, he was extolling. "If you wish to rid a man of melancholy thoughts, or of suicidal feelings, to remove from his breast envy, hatred, malice, and all uncharitableness, you must give him that article." This was but another way of telling us to cure the man's dyspepsia; the article alluded to being, in his estimation, the most efficient agent. There was much truth in the remark.

In discussing the important subject, of the best means of preserving the health of Ministers of the Gospel, I shall confine my attention to those diseases to which clergymen are especially liable. They are two in number—one of them, a form of laryngitis known commonly by the name of 'the clergyman's sore throat,' 'bronchitis,' &c., being but little known out of the ranks of their profession; the other, dyspepsia, shared by them in common with the members of the other learned professions, and with literary and sedentary men generally. This, I presume, is all that is expected. To advance beyond this, one could not well stop short of a complete treatise on hygiene; and if there is any other disease to which Ministers of the Gospel are peculiarly liable, or not shared by them in common with the whole human family, my memory does not now recall it; consequently, for their protection against all other diseases, the ordinary rules of hygiene are available.

First, of Laryngitis—*the laryngitis of clergymen.* It seems to be the impression of some that this disease is equally common among all classes of public speakers. I have rarely seen it except in Ministers of the Gospel. Were public speaking the whole cause, of course it would be equally common with all public speakers, in proportion to their exertions in that way. This, however, probably only predisposes, another element being necessary for its development.

To understand the nature of any disease sufficiently well to treat it philosophically, or to lay down any rational prophylactic rules, it is important that the causes leading to that disease, and the pathological change constituting it, should be understood. There are three causes which I suppose to have a paramount influence in the production of this disease: First. The overexertion or straining of the vocal organs in the effort of clergymen, in some instances, to make themselves distinctly audible to the most remote member, in others, the same straining of the voice beyond what is necessary—a vociferous energy with which they manifest their zeal for the cause in which they are engaged, apparently at the time forgetful of all else.

Another cause is, the sudden vicissitudes of temperature to which the clergyman is exposed, in passing in the winter season from the heated atmosphere of his study into the open air, thus in a few seconds breathing atmospheres differing many degrees in temperature. This, however, would not be so hurtful, were it not for the fact that the constant confinement to his room, which the nature of his studies, and his routine habits of life, to an extent require and encourage, renders him much more susceptible to injurious impressions from these vicissitudes. The unnecessarily high degree, too, to which many of them keep their rooms heated, the closeness of the apartments, and the injudicious care with which anything like free ventilation is guarded against for the purpose of preserving this high temperature, by which an excited state of the cutaneous exhalents is induced, still more strongly predisposes the system to be injuriously impressed by the sudden application of cold; from which result immediate suppression of perspiration and its consequent evils.

A third cause, composed of the two preceding, which it is probable has the principal agency in the production of this affection, and which so frequently directs the morbific effects of cold upon this part of the vocal apparatus, is the passing from heated churches after preaching, into the cold atmosphere, while the larynx itself and the neighboring parts are in—if the expression is not a solecism—a state of fatigued excitement and congestion, thus exposing them to a sudden change of temperature at a time when they are less able to resist its action. It is this state of fatigued excitement, I say, of the vocal apparatus, that gives a peculiar feature and location to the morbid actions excited by sudden vicissitudes of temperature. This view of the matter is supported, I think, by the fact that the upper part of the pharynx, the tonsils and palate, and all the parts immediately connected with the larynx, and which more or less partake of the actions going on in it, are almost equally with it exposed to sudden changes of temperature under the circumstances alluded to, will also be found to partake with it of the diseased actions induced by these causes. It may be said that the functions of the parts named, are so different from those of the larynx, that however intimately connected by location, they could hardly participate in the movements necessary to produce fatigue, determination to, or excitement in it. To this I could only answer, that the redness and engorgement of subacute or chronic inflammation, are always, so far as my observation extends, to be found in these parts when the disease in question is present in the larynx; and that in all cases where fatigue is at all experienced after declamation, these parts participate in the sensation.

Among that class of clergymen belonging to the Methodist church, denominated 'circuit riders,' whose duties are such as to require them to be much in the open air, in their visits to meet their different and distant appointments, if I am not mistaken, the disease in question is very rarely known. This is the case, at least so far as my own observation extends, and if it should be found to be a fact of general occurrence it goes far to support the view here advanced of the origin of the disease. To the exciting causes of the phlegmasiæ in general, the gentlemen in question are more exposed than other clergymen, and are probably with equal frequency the subjects of them; but spending much of their time in the open air, seldom being able to have their temporary apartments so highly heated, and the fresh air so completely excluded, as from the comfortable apartments of their stationary compeers, their systems are less predisposed to unfavorable impressions from the ordinary exciting causes of disease. But that which above every thing else renders them less liable to this disease, is that they seldom preach in churches in which the air is kept at a high temperature; it being a fact of general observation, that the country churches, or meeting-houses, in which the labors of these gentlemen are principally performed, are less carefully heated than those of the larger towns and cities. This being the case, on passing from the church, while the vocal organs are fatigued, and therefore predisposed to disease, they enter into an atmosphere at a temperature to which they are accustomed, and but a few degrees below that of the church in which they have been. Here then is a difference between stationary clergymen of the cities and larger towns, among whom laryngitis is common, and their compeers of the circuit, among whom it is almost unknown. The former after preaching pass from a high temperature into a much lower one, to which they are but little accustomed; the latter from a less heated atmosphere into one to which they are habituated. The same difference to a greater or less extent, obtains between the stationary clergyman and almost all other public speakers.

The causes then which peculiarly favor the development of laryngitis in clergymen, are, 1st, overexertion of the vocal organs; 2d, the vicissitudes necessarily incurred, and the predisposition induced by much confinement to highly heated apartments; and 3d, the sudden exposure of the larynx to the passage of air many degrees of temperture below that just immediately before respired, at a time when the previous exhaustion of the organ has rendered it less capable of resistance.

It is a subject of daily observation, that many clergymen, in their zealous enthusiasm, fatigue and exert their organs of voice to a point far beyond what is necessary to render their discourse distinctly audible to the persons in the church the most remote from their position. As to whether a discourse delivered in so high a tone, and with the degree of physical exertion generally accompanying it, may be more effectual than one otherwise delivered, it is not my present duty to give an opinion—but this I may observe, without wandering far from my subject, that the low, sweet, persuasive tones of some to whom I have listened, have seemed much more impressive. At all events, this laborious exertion of the voice is one of the most active causes of the disease of which I am treating; and as such, should as much as possible be guarded against by any one at all predisposed to laryngeal disease. To one who wished to make this effort, a friend seated near, say in the pulpit, by his side, would be useful by giving intimation when he was exceeding the proper tone. This would be found still more the case, if the elevation of his voice were the consequence of that forgetfulness of everything else—even of ones own personal good, to which many are liable when deeply interested with their subject. It cannot be doubted that a little restraint exerted in this way would have a salutary influence. In many instances too, this loud speaking depends upon a habit, imperceptibly and unintentionally fallen into. The same plan, aided by some exertion of will on the part of the subject of the habit, will be the most convenient means of correcting it. A little more attention to the principles of acoustics, in the construction of our churches, too, would have a beneficial influence, by rendering a high tone of voice less necessary. Do not the heavy cushions and rich carpeting of our city churches, increase the amount of exertion necessary on the part of the minister, by absorbing a portion of the sonorous vibrations? The adaptation of the size of the church to the voice and physical ability of the preacher of course cannot be always effected—nevertheless, as far as possible it should be attended to. It would be grossly violating the rules of clinical hygiene, if I may be allowed to coin an expression, to place a man of delicate frame and feeble voice, where his ministerial duties would have to be performed in a room, so large as to render great exertion on his part necessary to make himself heard in the remote seats; and to place another of herculean proportions and stentorian voice, to preach in a house so small, that his resounding tones would almost deafen his auditors:—and yet this is sometimes done. Churches cannot always be adapted to preachers, nor yet preachers to churches. A word or two on the latter. Any exertion of voice, above what is absolutely necessary for distinct audition, is to many ministers hurtful; and as it is unnecessarily so, should be more carefully guarded against. By attention to the matter, a minister may readily discover the degree of exertion necessary to render himself distinctly audible in any church, in which he may be called habitually to preach, and by care may, in a very short time, so accustom himself to it, that without effort he will readily and naturally fall into it. To aid in this intention, a friend may be stationed for a few Sabbaths in a distant part of the church, who, by some preconcerted signal might inform him as to whether he rendered himself distinctly audible, or spoke unnecessarily loud. The over fastidiously righteous, but less informed, might cavil at this. But is there any reason why Ministers of the Gospel should not adopt any innocent means for the preservation of their health? On the contrary, it is incumbent on them, above all men to do so. It is their duty to themselves, and to their flocks, which suffer from any incompetency of their pastors, by whatever cause produced.

The mode of removing the second of the causes enumerated above is obvious enough, though perhaps not so easy of accomplishment. In cold weather, the comfort of a warm room is so great, that its privation seems almost too much of a sacrifice to make for the prevention of a remote or possible evil. Much of this, however, depends on habit; and there are very few persons who do not keep their apartments in cold weather, at a temperature many degrees above the lowest point that would be equally agreeable, and much more conducive to health, were they gradually to accustom themselves to a lower temperature. As it is not the high temperature itself of the apartment, so much as the *difference* between this internal atmosphere in which the greater part of their time is spent, and the external atmosphere to which they are occasionally exposed, which proves injurious, the best method to guard against its action, is to keep the apartment habitually occupied, as few degrees above the external temperature as is at all compatible with comfort. This might be effected so gradually, as to render any great sacrifice of present comfort unnecessary, and indeed it is important for the health, as well as for the comfort of the individual making the attempt, that this reduction of the temperature of his apartment should be gradual. Let those who have been accustomed, then, to overheated apartments pay attention to discover the temperature most congenial to their feelings, and let them daily, or at longer intervals as may best suit, carefully attend to having it reduced a little: and for this purpose, the aid of a thermometer would be almost indispensable. By very gradually diminishing the quantity of fuel consumed, in a short time, without being at all unpleasantly affected by the change, one finds himself equally comfortable in a room, the temperature of which is many degrees below that to which he has been accustomed, and which he thought almost indispensable to his comfort. It is a fact, too, that many persons while engaged in study, frequently, through forgetfulness, or having their minds at the time deeply occupied, allow their apartments to become heated to a degree far above what is comfortable to themselves. This of course is more injurious, and should be particularly guarded against.

On the method of warming the apartment, but little need be said. The stove and the open fire-place, each has its advantages over the other, each its disadvantages. With the stove a more equable temperature of the apartment may be maintained with less trouble—while it has the disadvantage, among others, in a much stronger degree, of dessicating the atmosphere too much for a healthy respirable state. This may be obviated by evaporation from a vessel of water seated on the stove. The inconvenience from the difficulty of keeping up an equable temperature, in an apartment heated by an open fire-place, is perhaps overbalanced by the circulation of the air it induces, and the aid it thus lends to ventilation. This air, however, is not so great, in the usual construction of our fire-places and chimneys, as is usually supposed, as it is the lower cool and less vitiated strata of air to which the heat of the fire gives an upward impulse through the chimney, and not the more rarified strata above. I have somewhere seen it suggested to have an opening from the room, near the ceiling, communicating with the chimney. This would certainly be an advantage. Either for the stove or open fire-place wood, on several accounts, seems preferable. Its principal advantage, however, is probably owing to the smaller quantity of dust, or ashes produced from it, of sufficient levity to prove troublesome, by floating in the atmosphere. This is again measureably counterbalanced, by the less attention which a coal fire requires for keeping up a regular temperature. Upon the whole, the advantages of the stove and open fire-place, and of wood and coal, as fuel, are more equally balanced than persons exclusively accustomed to either will be brought readily to admit.

While this strict attention is paid to the temperature, equal attention is also required to the ventilation of the apartment. One thing should be carefully guarded against, in any process that may be adopted for the purpose; that is, that no current of air be allowed to blow directly on the occupant; and more especially on a limited part of the body. A double advantage arising from a lower degree of temperature of the apartment habitually occupied is, that it admits with less inconvenience of a more perfect ventilation. This is a matter of considerable importance, and is too much neglected. To admit a necessary supply of fresh air into a small apartment for its effectual ventilation, and with the least danger and inconvenience, the upper sashes of the windows should be so constructed, that by means of weights and pulleys they may be lowered or elevated at pleasure. The upper sashes of two windows, one on each of two opposite sides of the room may be lowered a few inches, more or less, according to the existing temperature of the room, and the sensations of the occupant. The elevated position of the opening for ventilation has the advatage of preventing anything like a direct current of air from blowing on the occupant. While by its greater condensaticn and specific gravity, a portion of the newly admitted air gravitates to the lower part of the room, displacing and pressing upwards the heated and contaminated strata. Another portion, may, when any current exists, be supposed, by the impetus it has received from without, to preserve its direct course, carrying out with it, that which has been displaced from below. When no current of force exists, there is merely a gradual admission of fresh air from each window, by which an equivalent portion of the strata below is as gradually displaced. To prevent any injurious effect from obliquely downward currents of air, which entering from above might impinge directly on the occupant of the room, a piece of board, a few inches broad, extending from one side of the window to the other, might be so attached to the upper part of the sash, with its inner edge obliquely upwards, as to give to the entering current, that direction. A still more efficient ventilation may be secured by having the opening for the admission of fresh air near the floor, and that for the exit of the vitiated air near the ceiling. By this means the fresh air on entering occupies its natural position, without having to gravitate through, and consequently become commingled with that which has been already vitiated. As it becomes heated and contaminated, without any opposing obstacle, it more readily takes its natural upward course, to be again succeeded by fresh portions. The openings of course, had better be on opposite sides of the room, that for admission facing the usual current of air. This latter method is attended with the inconvenience, without some care, of permitting currents of air to blow directly upon the occupant. This may be measurably, if not entirely obviated, however, by placing a screen, made so as to present a concavity to the entering current, between the opening and the seat occupied. The admission, however, of a sufficient quantity of fresh air for adequate ventilation is incompatible, at least without much inconvenience, with the high degree of temperature alluded to.

It is true that the habitual occupant of badly ventilated apartments becomes so accustomed to the foul atmosphere, that he suffers much less immediate inconvenience from it than one for the first time entering it, whose sensibility has not been blunted, or whose system not accustomed to it. The prisoner who has for a long time occupied his narrow cell, becomes unconscious of, or ceases to be unpleasantly affected by, the thick noisome atmosphere which at first seemed almost irrespirable, but still its poisonous influence is not the less exerted for being unperceived.

Although at no time should strict attention to ventilation be neglected, it is after meals, when the weather is such as not to admit of out-door exercise, that its importance is most imperative. This arises from the relationship, and to a certain extent, the mutual dependence on each other, of the functions of digestion and respiration, generally admitted by physiologists, but more fully and beautifully explained ot late, by Liebig, and others in the same field of research. As for the preservation of a healthy state of the system, a certain relation must exist between the ingesta, or rather the carbon and hydrogen contained in them, and the respired oxygen, it follows that a necessity for an additional or increased supply of the latter will be felt after each meal. Now, as the quantity of oxygen contained in a given volume of atmospheric air increases with the density of the air, and that again with a diminution of its temperature, the volume of air at each inspiration of any one individual, being as a general rule about the same, it follows that a pure and cool atmosphere is at this time especially important. Indeed the natural heat of the body thus generated by a due supply of oxygen for the combustion of the carbon and hydrogen of the food, during and after healthy digestion, will diminish the necessity for artificial heat. A healthy condition of the apparatus of digestion and assimilation, by which the food is more readily reduced to the condition favorable to the combination of certain of its constituents with the oxygen, favors the same end. Nothing improves the appetite so much as exercise in a cool pure atmosphere; nothing so well enables the stomach to digest that which the appetite calls for, as the same. In this the reciprocal action and dependence of the functions of digestion and respiration are exemplified. So beautiful, apparently so philosophical and consistent are the explanations of Liebig, that one almost sighs to think of the possibility of their being superceded by new developments, in the onward march of chemistry and physiology.

In the present day it would almost seem a work of supererogation to multiply examples of the baneful effects of inat tention to ventilation—one, however, may be admitted. I take it from a review of the report of the poor law commissioners on an inquiry into the sanitary condition of the laboring population of Great Britain, in the Medico-Chirurgical Review for April, 1843. "A large pauper school attracted great attention, by reason of the great mortality prevailing in it, and which was at once attributed to defective nourishment. On a searching inquiry, however, being instituted, the diet was found to be unusually good, but due ventilation wholly unprovided for. This was remedied, and in the same space where 900 children were by illness awakening extensive sympathy, 1100 now enjoy excellent health."

The remarks relative to the temperature of the study of a clergyman apply with increased force to the church in which he is in the habit of preaching, as it is in the transit from this to the open air, that his health is more in jeopardy, from the above mentioned causes, than from his home apartment. The church, then, should also be kept as little above the external temperature, as is at all consistent with the comfort of the congregation, while equal attention must be paid to its proper ventilation. In churches this can readily be effected without inconvenience or danger, as it may be done during the week or in the intervals between sermons; and unless the temperature is allowed to rise, during preaching, above the proper standard, there will seldom be a necessity for ventilation during this time. Should it, however, become necessary for the safety of the congregation, to admit fresh air during the service, as sometimes will happen with every attention, from the occasionally crowded state of the church, it would have to be accomplished by lowering the top sashes, instead of elevating the lower ones; but in a part of the church so remote, or so situated as to allow no direct current to the pulpit.

To prevent the evil consequences of the vicissitudes necessarily incurred, clergymen would do well to accustom themselves as much to the open air as the nature of their calling will admit. This will place the city clergymen in this respect, to a certain extent, in the position of his co-laborer in the country, where habituation to the free air of heaven, as I have already observed, renders him less liable to laryngitis.

Could any means, convenient and of easy application, be devised by which the change of temperature to which the fauces and larynx are exposed in the state of fatigue and predisposition to disease, in which they are after preaching, might be effected gradually instead of suddenly, it would undoubtedly be found beneficial. To a limited extent this may be accomplished by gargling the throat well with water at a temperature holding a medium between that of the church and the open air. To the water a few drops of tincture of myrrh may be added with good effect. In addition to this, where there is a vestry room, or an apartment of any kind immediately connected with the church, it might be kept at a proper temperature, and occupied for a short time after preaching, before passing into the open air.

The daily habit, which some probably neglect, of bathing the whole neck, the under surface of the lower jaw and throat externally, with cold water, will be found serviceable. To this friction with a coarse napkin may be added.

A cold shower-bath, of the shortest possible duration with the delicate, however, followed by friction with a coarse napkin or flesh brush, to the whole surface of the body, will be found doubly beneficial, in rendering the system less susceptible to the influence of cold; and, in consequence of the close sympathy between the stomach and the skin, by preserving the latter in a healthy state, and by the vigor and tone communicated to the system generally, warding off dyspepsia. The best hour for taking it, is probably in the morning, a short time before breakfast, and in cold weather it should not be taken without having a warm room to go into immediately after, as mischief would undoubtedly follow, unless immediate reaction succeeded. Those of a delicate constitution not accustomed to it had better commence with the tepid bath, and gradually reduce the temperature, especially if the habit should be first adopted in the winter season.

Much has been said of the advantages derived both by dyspeptics and persons predisposed to pulmonary disease, under which in common parlance is included laryngitis, from the constant wearing of flannel next the skin. They have probably been overrated, I would almost say absurd, when directed to be worn during the heat of summer. An uncomfortable degree of heat in summer is in all probability as prejudicial, as an uncomfortably cold temperature in the winter. But we are sometimes told that flannel is cooler in summer than linen. Is it possible that he who first said it, from whom the dogma has been echoed and repeated by thousands, had the natural healthy sensations? The overexcited state in which the cutaneous vessels is kept by it, when acting in combination with the heat of summer, must certainly predispose to catarrhal and other inflammatory affections, on the setting in of cold weather. A much more judicious course would seem to be, to adapt the clothing and its changes as nearly as possible, to the temperature and changes of the season—flannel for winter—cotton for spring and autumn—and linen for summer. Is it necessary to say here that the flannel should be frequently changed?

Although not required, a word or two on the *treatment* of the disease while in its incipiency may not be deemed inappropriate. For this purpose the external friction, above suggested, will be found beneficial, probably more so, in the early stage at least, than any powerful revulsive, as it may be kept up more permanently, and regulated better; and when combined with the cold bathing it seems to have the effect of giving tone to the neighboring parts, while it allays the irritability of the nerves. I was led first to recommend it for this purpose and thus combined, from witnessing its beneficial influence in persons liable to frequent attacks of quinsy; and in others habitual sufferers from toothache and other painful neuralgic affections of the face. The use of astringent and slightly stimulating gargles, when used in moderation, will in almost every case be found serviceable. They should be used cool, or cold. For this purpose, a weak solution of alum, say five or ten grains, to the ounce of water, to which may be added a small quantity of tincture of myrrh, will be found as useful as any. But that which above every thing else seems to have the most decided influence in arresting the progress of the disease, is the occasional application to the palate, tonsils, and all that portion of the pharynx of easy access, of a solution of nitrate of silver, say ten grains to the ounce of water. In the more advanced stages, a much stronger solution will be necessary, or perhaps the caustic in substance. As the use of the solid caustic is always attended with some little danger, its application should be entrusted only to the hands of a physician. A solution, however, or rather a paste of a drachm of the caustic to half the quantity of water, may with perfect safety be applied with a small mop, and is probably equally as beneficial as the caustic in substance. It will be seen that I have not spoken of its application to the part principally implicated, and from which the disease has its name. This may be done by the physician, in the manner directed by Trousseau and Belloq in their work on laryngeal phthisis. I cannot but think, however, that the case must be a serious one indeed, that could justify a resort to the application of caustic and astringent solutions and powders to a part, to which the accidental application of a drop of the most bland fluid, causes at times so much inconvenience. I have myself never found it necessary to resort to any application to the glottis or larynx itself—the action of the remedy, applied as directed, seeming by continuous sympathy to extend to, and produce the desired influence on the part. I can, however, conceive it to be more than probable that in cases of ulceration of the larynx, the immediate application of the caustic would be absolutely necessary to produce to the full extent its peculiar influence.

So much has been written upon the subject of dyspepsia, so many different "cures" recommended or suggested, so many different opinions advanced, that as proteiform have become the essays upon it, as are the manifestations of the disease itself. I have nothing new to offer on the subject, but will attempt, as briefly as possible, to enumerate the causes which are more especially concerned in its production among clergymen, and to suggest the means of avoiding them.

First. Want of the due amount of exercise, regularly taken, and at proper times. Almost every man whose calling is such as to require sedentary habits, neglects to take the amount of exercise necessary for the perfect preservation of his health, and thus, by the dullness of intellect, and the incapacity for mental exertion brought on in this way, loses more time than would have been necessary to preserve his health if spent judiciously in exercise. One hour of mental labor, when the mind unembarrassed by disease and uncomfortable bodily sensations concentrates itself wholly upon its subject, being more productive, than twice the time spent in efforts to direct attention to a topic under considertion, while obscured by the clouds arising from an undigested meal, and distracted by headache, and the other attendant evils of dyspepsia. To the proper amount of exercise, then, taken at appropriate times, I would direct attention, as a matter of the first importance. As to the kind of exercise, there is none so beneficial as walking. I may modify this remark, however, so far as to state, that when any laryngeal inflammation already exists, equestrian exercise will be found preferable, or at least a principal portion, the most active part, should be of this kind.

I will suggest the exercise for a day, such as I suppose may be most generally applicable, liable of course to variations and modifications, to suit the different powers and capacities of different persons. Early rising and exercise before breakfast have been much lauded, by those perhaps who have not made the trial, and the system looks very well on the pages of a romance. To the first, of course there can be no objection, nor to the latter, but not carried to the extent generally recommended. Breakfast should be taken very soon after rising. A short walk, before breakfast, in the open air, not carried so far as to produce heat, or but the slightest degree of fatigue, is as much as would be proper. After breakfast, from an hour to an hour and a half may be spent in a walk in the open air, or some easy labor, such as working in the garden. The exertion should not be such as to produce much fatigue, especially during the earlier part of the allotted time. From this till within half an hour of dinner, the time may be spent in reading or in composition; the half hour immediately preceding dinner, in a gallop on horseback, or in some other appropriate exercise, but if carried far enough to produce fatigue, a period of rest should intervene before dinner. As dinner is probably the meal most productive of dyspepsia, the period of its digestion is the one to which attention should especially be directed. In this respect there is considerable diversity of opinion among authors, some, a few, recomending during its digestion, active, perhaps, laborious exercise; some a state of perfect quiescence, and others a medium course. The latter in the greater number of cases will be found most beneficial. The first hour after dinner should be spent in gently promenading the room, in bad weather; or, in more favorable weather, walking at an easy pace in the open air. After this, the amount of exertion may be increased, and after the process of digestion may be supposed to be nearly complete, say in two or two and a half hours, the degree of exertion may be still further augmented, and if exercise carried to fatigue is at all beneficial, now is the appropriate time. After an hour's active exercise, followed by half an hour's rest in the recumbent posture, for above all things, men who study or write should reserve the sitting posture for these purposes, the labors of the day may be resumed. To his out-door exercise, as constituting an agreeable and useful employment for the mind, it being also calculated to remove any impression of time wasted, attention may be paid to botany, gardening, horticulture, &c. Half an hour or an hour should be spent in exercise before tea, and it would be well to spend the same time in a gentle promenade after tea, before resuming the pen or book. The night should not be so far infringed upon, as not to allow of sufficient time for repose; and again an hour's exercise, within doors, or in the open air, asthe weather may or may not favor the latter, before retiring. My recollection at this time presents before me the cheerful and enlivened faces of two clergymen, one of the Presbyterian, the other of the Episcopal church, who in the opinion of some almost outrage the gravity of their order by the rapidity of their pedestrian movements. The effect is manifested, though neither is of a robust constitution, by the ruddy glow, and sparkling eye of health, with which their countenances are animated.

Another fruitful cause of indigestion is the constrained position, unavoidable to a certain extent, which persons assume while engaged in study or composition. The bent-forward position, into which almost every person unconsciously falls while writing, in which the chest and diaphragm press heavily upon the abdomen, constraining and embarrassing the stomach in its gentle motions during digestion; and the impediment thus produced to the free passage of the chyme through the pylours from the stomach into the duodenum, the same pressure upon the bloodvessels contained in the abdomen, more especially the vena cava, preventing the easy return of the blood to the heart, and thus producing a state of engorgement of the abdominal viscera generally, must have a powerful agency in the production of indigestion. This may be avoided to a certain extent, by an individual engaged in study accustoming himself to read in the erect position, at the same time gently pacing his apartment. The mind might not at first be so easily concentrated, but custom would soon obviate this. Besides, almost every literary man, clergymen perhaps among the rest, do a certain amount of reading which does not require great exertion of intellect. This portion of their reading only might first be performed in this manner. Much of one's writing might also be accomplished in the erect position, as is the case with most of the book-keepers and clerks, in mercantile establishments; and if at first, any difficulty should be experienced in directing attention to the subject, only the less intellectual parts, such as copying, might be performed in this way. By some attention to the above suggestions, less necessity will be felt, and less time required for exercise in the open air.

The subject of diet next engages attention. Here I shall be brief, for were I to attempt to set down all that might be considered important relative to it, it would swell the present article, to perhaps an unreadable extent, and consequently frustrate the object of my labor. Without, therfore, entering to any extent into theoretical discussions relative to the materia alimentaria, I shall merely suggest a few simple rules, or rather hint at a few points, attention to which appears to me of most importance. Of these, moderation in eating stands pre-eminent. By this I do not mean starvation, or something bordering upon it, as some who carry everything to excess, would construe the caution, but a due supply of wholesome nutriment. To insure the proper quantity, and no more, being taken, a longer time than is usual in this country should be spent at the table, to give the *stomach an opportunity of appreciating the ingesta*, and to admit of slow and effectual mastication. Though I cannot fall into the opinion of those who advise that the meal should be discontinued while a sensation of hunger is still felt, yet, anything like a feeling of repletion or satiety, should be avoided. Large quantities of fluids during the meal, and after, until the process of digestion is complete, should be avoided, as tending to distend the stomach beyond the point at which it grasps, and most effectually contracts upon the alimentary mass, and also as tending to dilute the gastric juice, so that its solvent properties are but inefficiently excited. For the latter reason, also, they should not be taken freely immediately before eating. Or should we adopt the theory of Liebig and others, that the process of digestion is one of transformation of the food, a new arrangement of its particles in consequence of contact with other particles undergoing at the same time the same transformation, as in the process of fermentation; these other particles, which are supposed to communicate the impulse in this transformation to the alimentary mass, being contained in that fluid called the gastric juice, its dilution may be equally supposed to affect their power. The fact, however, is unalterable, though theories or explanations may change.

As for the much abused tea and coffee, a *good* cup of either, may be innocently, if not benefically, indulged in at supper or breakfast—the injury, if any, arising from the use of these articles, being from the excessive quantity of the *menstruum*, and not from the ingredient, acting as above suggested in too much diluting the gastric juice. It is at dinner, as being the principal meal, and the one generally of most difficult digestion, that a free indulgence in fluids should be avoided. It is true, that the '*bon vivant*' indulges freely in fluids during and after dinner, but still they are more stimulating in quality, than excessive in quantity, and are at the time no doubt useful, from their stimulating properties, in enabling the stomach to master the unnatural load with which it is overburthened. This organ, however, seldom fails in the end to be avenged upon the system generally, for any bad treatment imposed upon it by the palate.

Although the proteinaceous compounds, albumen, casein and fibrine, constituting the elements of nutrition for the body, are identically the same in vegetables and animals, those of the latter indeed seeming only to be derived from the former; still experience proves, that a mixture of animal and vegetable food is better for the preservation of health, than the exclusive use of either one or the other alone; the form and combination, and the degree of concentration or dilution of our articles of diet, probably modifying their amenability to the action of the stomach. Indeed, as a general rule, it is a fact, that the very elements of nutrition themselves, in a state of concentration separately, are very unfavorable to the support of animal life; and Magendie mentions the fact of animals dying of inanition in two months, though consuming daily from one to about to about two and a half pounds of fibrine. Of albumen alone, "after a few day's use of it, they refuse to take it, preferring to suffer the most violent pangs of hunger, rather than eat it, and ultimately they die of inanition."

The state of combination of the elements of nutrition, as found in some animal substances, as in beef, mutton, &c., seems in many instances to be most agreeable to the stomach of the dyspeptic. A quantity of vegetable food is important however, probably, for the supply of another proteinacious principle, gluten, very nutritious, and capable by itself, for a long time, of supporting animal life, and for a supply of a sufficient quantity of the non-nitrogenized components of our food, '*the elements of respiration.*'

A due mixture of animal and vegetable food, then, is important: to which should be added at times, a share of good ripe fruit, for reasons hereafter to be mentioned; those of easy digestion, however, being carefully selected, while the cores, skins, seed, &c., are to be rejected. It is a common but mistaken opinion, that these parts render fruits more easy of digestion, and I was once asked, why nature so produced them if not to be eaten. As well might it be asked why nature produced the oyster with a shell, if it was not intended that it should be thus eaten. Like the pickles, the condiments, and stimulating drinks of the epicure, although they can hardly be said to render the substances themselves more digestible, by their irritant qualities they may excite the stomach temporarily, so as to enable it to digest in undue proportion. It is a morbid excitement, however, and never fails when carried far to be followed by a proportionate state of languor, or debility of the organ. As regards condiments, their use should be, as a general rule, as much as is consistent with a palatable state of the food, avoided. Persons of a robust, plethoric habit, or sanguine temperament, need them not, and to those of a nervous or bilious temperament they are frequently injurious, by exciting the appetite to an improper indulgence, and producing in the stomach itself a state of inflammation, or one of irritation bordering upon it. If to any a free use of condiments is beneficial, it is to those of a leucophlegmatic habit of body. With them, the tone of the stomach is generally weak, while stimulants of the kind are less liable to irritate or inflame. But in regard to diet, no fixed and invariable rules can be established, for as long as there are differences in the physical proportions, in the temperaments, constitutions, and habits of men—so long must there be a certain degree of latitude allowed in dietetic regulations. Each man must be governed to a certain extent, in his own case, by his own experience. This to men of judgment and observation may safely be entrusted. The stomach very soon gives intimation of bad or oppressive treatment, and if its first warnings are carefully heeded much future suffering may be avoided.

A constipated state of the bowels, in sedentary men, is one of the first evils to which their habits of life expose them. It is one of the first accompaniments of dyspepsia, and in all cases has a tendency to aggravate it. For its cure, or prevention, a regular system of exercise is above all things important. While allowing due attention to the first calls of nature, a certain degree of regularity in the periods may be established. This is principally beneficial as having a tendency to direct attention to the matter, and therefore prevent neglect. In many things, we are the 'creatures of habit.' This is not only the case in our voluntary acts, but in the series of organic changes, and instinctive movements, over which *will* has at most but an indirect and imperfect control. For the preservation of the bowels in a soluble state, a moderate indulgence in fruits has a favorable influence: their acid and saccharine juices acting as a mild, pleasant, and natural laxative, while the irritation produced by purgatives is avoided. They should be used, however, with the restrictions above suggested. The most digestible, and therefore the most suitable, are probably the apple and peach. There are some fruits very indigestible; the pine-apple is peculiarly so. It may be allowed, however, with the precaution of swallowing only the juice, and rejecting the substance of the fruit.

Shall I touch upon the use of medicine in incipient constipation? It should be as much as possible avoided—by every other means striving to keep an open state of the bowels before resorting to this. The use of purgatives once commenced, is very apt to become habitual, the torpor and inactivity following the excitement and irritation produced by one dose, almost always requiring another, and in augmented proportions. If medicine is at all resorted to, the smallest possible dose, capable of rousing the action of the intestinal canal, should be taken. For this purpose, a pill containing from one to three grains of aloes, half a grain of blue mass, and a grain and a half of extract of hyoscyamus, answers the purpose well. Unirritating lavements, however, may in many instances be substituted for even the mildest laxatives, without the risk attendant upon the latter, of rendering permanent by their use, any temporary derangement or irritation of the stomach, or constipation of the bowels, which may seem to call for them.

Will I be accused of a disposition to favor too much the medical profession by recommending persons laboring under a torpid or inactive state of the abdominal viscera, first to consult one of its members before resorting to the use of any 'specific,' or 'cure,' however highly recommended? In the present instance, I shall certainly avoid this, as my advice is to a class, to whom our services are willingly rendered gratuitously. The habit too frequently followed, so soon as the bowels become a little costive, of swallowing in quick succession, 'specifics,' 'bitters,' 'tonics,' 'cures,' &c., cannot be too much condemned; and yet thousands of cases of inveterate constipation and dyspepsia are thus induced. Is it not a fact that clergymen have a peculiar *penchant* this way? These gentlemen I know will pardon me if I wrong them, as it is for their especial benefit that I write, but from the number of advertisements of *specific cures*, with which almost every newspaper groans, the efficacy of which is *certified by them*, it would seem so, for certainly their certificates are not procured without trials of the remedies.

All authors who have written on the influence of the *physique* upon the *morale*, have dwelt strongly upon the power exerted by the digestive apparatus over the mental manifestations. The importance of a healthy state of the stomach, for the due exercise of the mind, is second only to that of the brain itself: no other part of the body seeming to have anything like so intimate a sympathy with the organ of the mind as this. How important then, that an organ exerting so decided an influence over the productions of the mind, should not be abused:—an organ whose healthy or diseased condition gives buoyancy, energy, and determination; or gloom, melancholy, irresolution, and imbecility to the mental phenomena, as the case may be; one whose diseased condition may be productive of every degree of depravation of intellect, from the slightest lowness of spirits, to the most violent insanity; one whose cravings under the influence of want or starvation, induces a state of the affective faculties in which man forgets his humanity, and levels himself with the beast of prey; in which ties the nearest and most binding are severed; in which love, friendship, and relationship are set at naught; in which the most violent antipathies of civilized man are overcome, and cannibal like he takes the life of his fellow in misery, to feed upon the quivering flesh of him who but a short time before was his companion, messmate, or brother; in which self-preservation, that most selfish of all man's instinctive feelings, becomes his only rule of action; nay, in which his own flesh is not secure, but in his savage and reckless desperation, is torn and mangled by his own reeking jaws. One tie alone, the tie that binds the mother to her infant offspring, has been known to withstand the cravings of famishing despair, alone amidst this exhibition of the savage brutality of man's nature, has stood as a redeeming trait. Her heart *alone* has been forgetful of all selfish cares; she has been known, when starvation has arrived at that point, against which her physical frame could no longer bear up, calmly to sink into the arms of the grim messenger, careful of, and to her infant offspring, supplying even in death, "nature's pabulum, from nature's fount."

